# Epigenetics and DNA Base Substitutions of Epstein–Barr Virus (EBV)-Related Gastric Cancers: Implications for Targeted Therapies

**DOI:** 10.3390/genes17070769

**Published:** 2026-06-30

**Authors:** Ioannis A. Voutsadakis

**Affiliations:** 1 Division of Hematology, Oncology and Blood and Marrow Transplantation, University of Iowa Hospitals and Clinics, Iowa City, IA 52240, USA; ivoutsadakis@yahoo.com or ivoutsadakis@nosm.ca; 2 Section of Internal Medicine, Division of Clinical Sciences, Northern Ontario School of Medicine, Sudbury, ON P3E 2C6, Canada

**Keywords:** stomach adenocarcinoma, viral, gamma herpesvirus, latency, methylation

## Abstract

Background: Gastric adenocarcinomas constitute a histologically and genomically heterogeneous group of cancers. The genomic classification of gastric cancers in four groups by The Cancer Genome Atlas (TCGA) has defined a framework for pathogenic discoveries. One of the groups is associated with infection by the gamma herpes virus Epstein–Barr virus (EBV) and represents a distinct subset of gastric cancers with potential therapeutic opportunities. Methods: The EBV-associated cancers from the TCGA gastric cancer cohort were analyzed to determine specific mutational and mRNA expression profiles that set these cancers apart from other gastric cancer subtypes. The cBioportal for Cancer Genomics site was used for downloading and analyzing the primary data. Results: EBV-associated cancers represented about 7% of the cohort. Mutations in the catalytic alpha subunit of PI3K kinase, *PIK3CA*, and the epigenetic modifiers *ARID1A* and *BCOR* were common. *PIK3CA* mutations were observed in 80% of EBV-associated cancers and frequently affected the hotspot codons E542 and E545. The few cases without *PIK3CA* mutations displayed frequent alterations in *ERBB2* or in the regulatory unit of PI3K. EBV-associated cancers did not display excess cytidine to thymine (C>T) transitions compared with other gastric cancer genomic subtypes, as would be expected from the high genome methylation caused by the virus. In contrast, an increased rate of T to G (T>G) transversions was observed in EBV-associated cancers. Translesion polymerase eta (POLH), which produces a signature characterized by a preponderance of T>G, was up-regulated in EBV-associated gastric cancers and may be a contributing factor in this increase, up-regulated by wild-type p53 and over-expression of transcription factor IRF1. Conclusions: The data presented here suggest that mutagenesis in the EBV-associated gastric cancers is not a direct consequence of the virus-derived hypermethylation. Up-regulation of kinase PI3K and its pathway is a prerequisite for EBV transformation, and epigenetic alterations are frequently present, suggesting therapeutic avenues.

## 1. Introduction

Gastric cancer remains a lethal neoplasm with high prevalence globally [1]. The landmark studies of The Cancer Genome Atlas (TCGA) identified four genomic subtypes of gastric adenocarcinomas: the chromosomal instability (CIN) subtype, which accounts for approximately 50% of cases, and three less prevalent subtypes, namely microsatellite instability (MSI)-high (16%), genomically stable (13%), and Epstein–Barr virus (EBV)-related (7%) tumors [2]. The four genomic subtypes present specific molecular and clinical features. The predominant CIN-high gastric cancers are characterized, as implied by their name, by a high burden of copy-number alterations, including amplifications of receptor tyrosine kinases and cell-cycle regulators. Frequent mutations in *TP53* are also present, supporting the role of p53 function in protecting from large-scale genomic gains and losses [3]. The MSI-high subtype is associated with MLH1 promoter methylation and a high tumor mutation burden (TMB). The genomically stable subtype correlates with diffuse histology and possesses mutations in the epithelial cell junction protein E-cadherin. The EBV-associated type is characterized by high levels of genomic methylation, which is the highest among any cancer type [4]. Remarkably, despite the high levels of methylation, the MLH1 promoter is not methylated, attesting to the site specificity of the epigenetic modifications. In contrast, the promoter of the cell-cycle inhibitor gene *CDKN2A* is frequently methylated, leading to loss of expression of cyclin-dependent kinase inhibitor p16. EBV-associated gastric cancers also show frequent mutations in the catalytic alpha subunit of the PI3K gene, *PIK3CA*, and in the epigenetic regulator *ARID1A* [5].

EBV infection of gastric epithelial cells leads to the entry of viral DNA into the cell nucleus, where it is established as a circular chromatin-associated episome [6]. The viral genetic program promotes the methylation of the viral genome to establish latency, and results concomitantly in extensive host genome methylation through up-regulation of host methyltransferases. The exact mechanism is not entirely clear, although it appears to involve the activation of both de novo (DNMT3A and DNMT3B) and maintenance (DNMT1) host methyltransferases [7]. Viral LMP2A protein activates cellular signal transduction pathways to mediate methyltransferase up-regulation [7]. LMP2A promotes carcinogenesis through activation of the PI3K/ AKT pathway [8]. In contrast, demethylation of CpG islands in gene promoters by the action of TET2 methylcytosine dioxygenase induces the activation of the viral lytic cycle [9]. Most viral proteins are not produced in the latent phase of the viral cycle of life, and this avoids detection from the host immune system [10]. Promoter methylation and suppression of transcription factor Interferon Regulatory Factor 5 (IRF5) by EBV down-regulates the host immune response against the virus and promotes oncogenesis [11]. The viral genome continues to be present in a latent phase during the progression of the EBV-associated cancers, although there have been cases where the cancer genome was cleared from some of the cancer cells in a tumor [12]. EBV is associated with carcinogenesis in both epithelial and hematopoietic cancers, which include, in addition to gastric cancers, nasopharyngeal carcinomas and Burkitt lymphomas, Hodgkin lymphomas, and others [13]. Although in all EBV-related malignancies the virus establishes latency to escape destruction of host cells and detection by the immune system, the specific mode of latency varies, and transcripts of the virus that remain expressed and permit transformation range in numbers between a few and several dozen [14]. This suggests that the specific type of host cells and their molecular infrastructure are critical for transformation. The current study examines the role of methylation and cellular responses to EBV-induced alterations in the genome of EBV-associated gastric cancers to reveal mechanisms involved in carcinogenesis that may have therapeutic implications.

## 2. Methods

Single-base substitutions were downloaded from the mutation files of the TCGA gastric cancer study provided in the cBioportal Cancer Genomics site [2]. For the genomic analyses of gastric cancer data, TCGA has used a whole-exome next-generation sequencing approach. Genomic data generated include mutations, copy number alterations, structural variants, and mRNA expression. Various pipelines were employed for nucleotide mutation calling [15]. mRNA expression from RNA-Seq data was performed in the TCGA series with an algorithm called RSEM (RNA-Seq by Expectation Maximization), which is able to process data without a requirement for a reference genome [16]. mRNA expression was calculated as a normalized mRNA expression z score compared with normal samples. Chromosomal instability (CIN) was measured by two scores, the aneuploidy score (AS) and the Fraction Genome Altered (FGA). The former was defined as the number of chromosome arms that had copy number alterations (gains or losses), and the latter was defined as the fraction of the total genome length with copy number gains or losses. Up-regulation or down-regulation of mRNA expression of transcripts of interest was defined as a normalized mRNA expression z score above 1 or below −1 compared with normal samples.

All analyses of the gastric cohort of TCGA and of the gastric cancer cell lines from the Cancer Cell Line Encyclopedia (CCLE) collection were performed using data hosted in the cBioportal for cancer genomics site (www.cbioportal.org, last accessed 13 May 2026) [17,18]. cBioportal contains series deposited from various research consortia and is publicly available and free to download for the broad scientific community.

The COSMIC (Catalogue of Somatic Mutations in Cancer) catalog (https://cancer.sanger.ac.uk/signatures/sbs/) of single-base substitutions was interrogated for identification of signatures with relevance in EBV-associated gastric adenocarcinomas (last accessed 14 May 2026). COSMIC contains signatures derived from genomic studies of various types of cancers, some of which have been assigned specific causative associations [19].

mRNA and protein levels of enzymes of interest in normal epithelium and gastric neoplastic tissues were examined in the Human Protein Atlas database (last accessed 14 May 2026) [20]. The mRNA expression data were either internally generated by the Human Protein Atlas or imported by the Genotype Tissue Expression (GTEx) project and were expressed in normalized transcripts per million (nTPM) of mRNA [21]. Protein expression data were generated by the Human Protein Atlas [20]. The antibodies used in immunohistochemistry sections were HPA064853 for REV3L, HPA044534 and HPA051036 for REV1, HPA006721 and HPA026762 for POLH, and HPA064696 for POLI.

The pathogenicity of mutations in genes of interest was assessed using data provided by the OncoKb knowledgebase (last accessed 17 May 2026) [22,23]. This knowledge base, hosted in www.oncoKb.org, is manually curated and publicly available, providing information on the pathogenicity of genetic alterations in cancers, analyzing a diversity of available resources. Evaluated mutations encountered in cancer cases are categorized as oncogenic, potentially oncogenic, or neutral. The databases used in the current article and their electronic addresses are summarized in Table 1.

Fisher’s exact test (mathematical expression P = [(a + b)!(c + d)!(a + c)!(b + d)!]/a!b!c!d!n!, where a, b, c, d are the values of a 2 × 2 table, n is the total number of observations, and ! is the factorial operator) or the x^2^ test (mathematical expression: Χ^2^ = $\sum \left[\frac{\left(O-E\right)2}{E}\right],$ where ∑ is the sum of all calculations, O is the observed frequency, and E is the expected frequency) was employed for the statistical comparisons of categorical variables and Student’s *t*-test (mathematical expression: t = m1 − m2/$\sqrt{\left(\frac{s1}{n1}+\frac{s2}{n2}\right),}$ where m1, m2 are the means of the two samples, s1 and s2 are the variances of the two samples, and n1, n2 are the sizes of the two samples) or Analysis of Variance (ANOVA) was used for the statistical comparisons of continuous variables. Correlations were examined with the Spearman correlation coefficient (mathematical expression: r_s_ = 1 − 6∑D_i_^2^/n(n^2^ − 1), where D_i_ is the difference between the ranks for pair I and n is the total number of observations). All statistical comparisons were considered significant when p was less than 0.05.

## 3. Results

**Figure 1:**
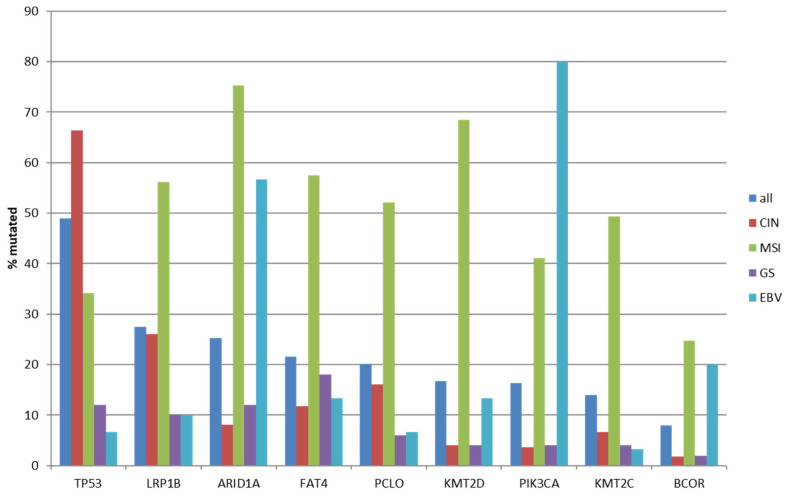
Prevalence of mutations in frequently mutated cancer-associated oncogenes and tumor suppressors in the entire gastric adenocarcinoma cohort (labeled all) and according to the four genomic subtypes. CIN: Chromosome Instability subtype, MSI: Microsatellite Instability subtype, GS: Genomically Stable subtype, EBV: EBV-associated subtype. Data are from The Cancer Genome Atlas (TCGA).

The stomach adenocarcinoma TCGA pan-cancer cohort contained genomic data from 440 analyzed patients. The four subtypes of gastric adenocarcinomas displayed significant differences in the prevalence of the most frequent mutations (Figure 1). The CIN subtype showed a high prevalence of *TP53* mutations (66.4%), which was higher than the prevalence in the entire cohort, across subtypes (48.9%), and almost double the prevalence in the next most prevalent group (MSI-high: 34.2%). The MSI-high subtype, which was the second most prevalent gastric cancer group, displayed a higher frequency of several mutations, including epigenetic modifiers *ARID1A*, *KMT2D*, and *KMT2C* and cadherin *FAT4*, all of which approached or surpassed the 50% of MSI-high cases (Figure 1). The EBV-associated subtype of gastric cancers is the least frequent and displayed a very high prevalence of *PIK3CA* mutations (80%), which was significantly higher than the overall prevalence in the entire gastric cancer cohort (16.3%). The MSI-high group of gastric cancers also showed a high prevalence of *PIK3CA* mutations exceeding 40%.

**Figure 2:**
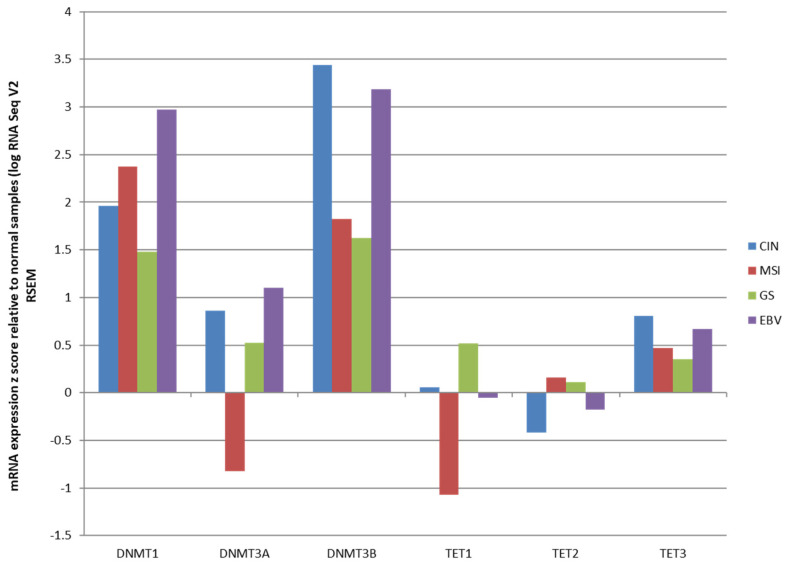
mRNA expression (mRNA expression z score relative to normal samples) of DNA methyltransferases (DNMT1, DNMT3A, DNMT3B) and TET demethylases (TET1, TET2, TET3) according to the genomic subtype of gastric cancers. CIN: Chromosome Instability subtype, MSI: Microsatellite Instability subtype, GS: Genomically Stable subtype, EBV: EBV-associated subtype. Data are from The Cancer Genome Atlas (TCGA).

The expression of both DNMT1 and DNMT3B DNA methyltransferases was up-regulated in all subtypes of gastric cancer, with CIN and EBV-associated cancers showing significant up-regulation of DNMT3B (mean mRNA z score relative to normal samples over 3) and DNMT1 approaching also a mean mRNA z score relative to normal samples of 3 in EBV-associated cancers (Figure 2). DNMT3A showed lower levels of up-regulation (mean mRNA z score relative to normal samples above 1) in EBV-associated cancers. TET family enzymes, which contribute to demethylation of methyl-cytosines and would counteract the activity of methyltransferases, were not up-regulated (Figure 2).

**Figure 3:**
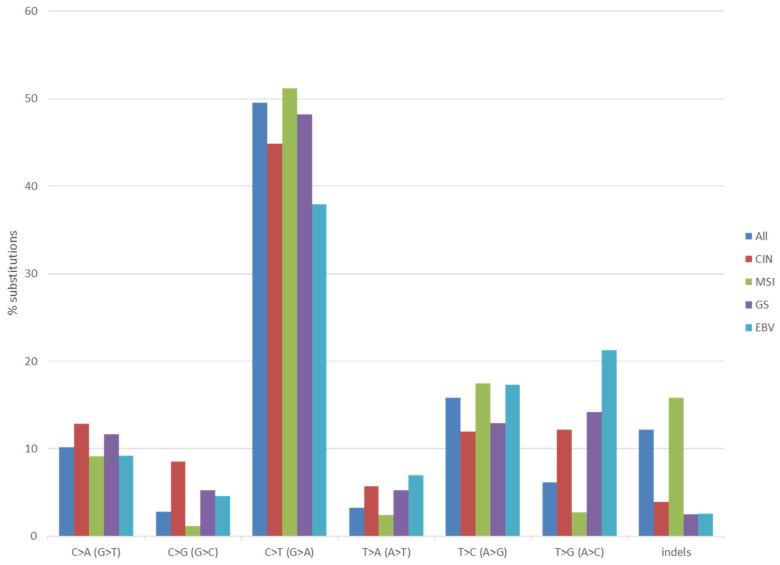
Percentage of specific types of single-base substitutions in the entire gastric adenocarcinoma cohort (labeled All) and in the subtypes of gastric adenocarcinomas. The conventional pyrimidine code is shown on the x-axis, and the substitution in the complementary chain is shown in parentheses. CIN: Chromosome Instability subtype, MSI: Microsatellite Instability subtype, GS: Genomically Stable subtype, EBV: EBV-associated subtype. Data are from The Cancer Genome Atlas (TCGA).

To explore whether the increased DNA methylation observed in EBV-associated gastric carcinomas was the underlying cause of mutations by increasing the frequency of methyl-cytosines, which are prone to undergo C to T (C>T) transitions, the prevalence of specific single-base substitutions (transitions and transversions) was examined in the gastric TCGA cohort (Figure 3). C to T transitions or the equivalent G to A transitions in the complementary chain were the most frequent type of substitutions in all gastric subtypes, as observed in other cancers. However, they were not increased in EBV-associated gastric cancers compared with other subtypes, confirming that the extreme hypermethylation observed in these cancers was not a direct cause of increased hypermutability. In contrast, the subset of EBV-associated gastric cancers had a higher prevalence of T to G (T>G) transversions or the equivalent A to C (A>C) substitutions (21.3% versus 6.1% in the entire cohort, Fisher’s exact test *p* < 0.001, Figure 3).

**Figure 4:**
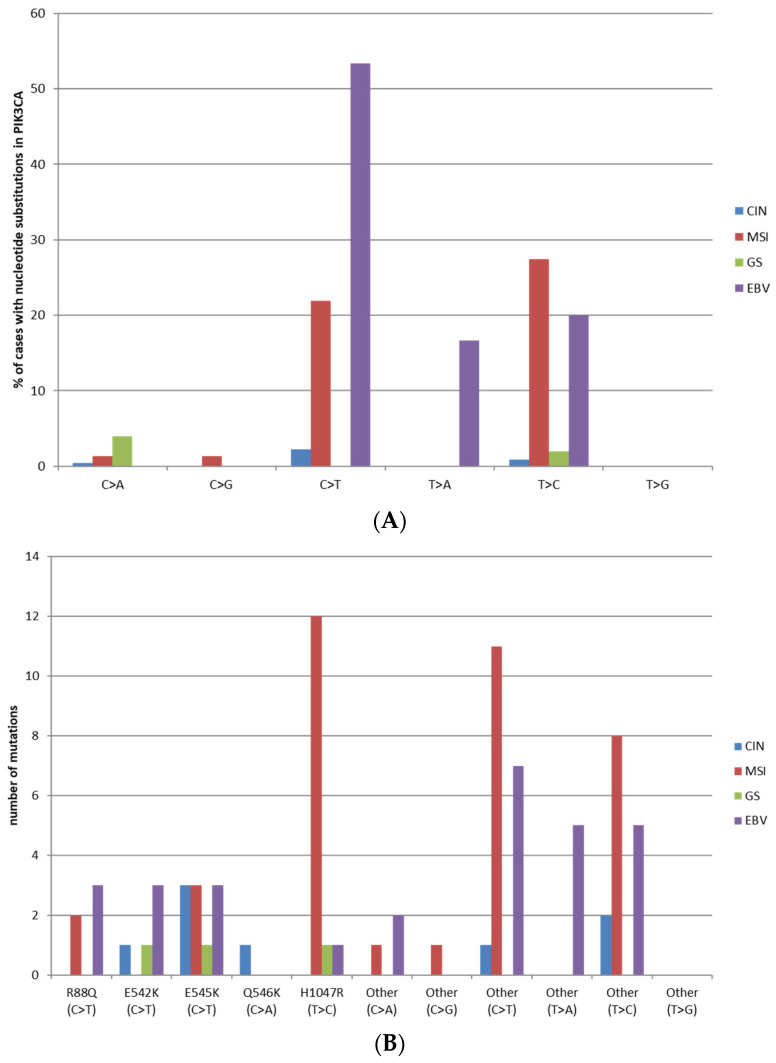
(**A**). Percentage and (**B**). Number of specific base substitutions in *PIK3CA* according to the subtype of gastric adenocarcinomas. In panel (**A**), the conventional pyrimidine code is shown on the x-axis. In panel (**B**), the conventional pyrimidine code is shown on the x-axis in parentheses below the respective nucleotide substitutions. Other denotes non-hotspot mutations that produce the respective substitutions shown in parentheses. CIN: Chromosome Instability subtype, MSI: Microsatellite Instability subtype, GS: Genomically Stable subtype, EBV: EBV-associated subtype. Data are from The Cancer Genome Atlas (TCGA).

Regarding mutations in the *PIK3CA* gene, the specific *PIK3CA* substitutions differed between the groups, with C>T substitutions predominating in EBV-associated cancers and T>C substitutions being the most frequent in MSI-high cancers (Figure 4A). T>C, as well as T>A substitutions, were also observed in lower numbers of EBV-associated cancers, and C>T substitutions were observed in a smaller subset of MSI-high gastric cancers. The number of mutations in *PIK3CA* observed in CIN and GS cancers was too low to derive any meaningful conclusions regarding the prevailing specific transitions or transversions. Among the hotspot mutations in *PIK3CA*, R88Q, E542K, and E545K, which are produced by C>T substitutions, were more prevalent in EBV-associated cancers, while H1047R mutations, which are produced by T>C substitutions, were most prevalent in MSI-high cancers (Figure 4B). C>T substitutions were also the most prevalent cause of non-hotspot *PIK3CA* mutations in EBV-associated cancers, which are predicted to be pathogenic, although T>A and T>C substitutions were also prevalent (Figure 4B).

Only five samples from EBV-associated cancers (20%) had no *PIK3CA* mutations in the TCGA gastric cancer cohort. Interestingly, three of these had amplifications (two samples) or structural variants (one sample) in *ERBB2*. These alterations were observed only in one case (4%) of EBV-associated cancers with *PIK3CA* mutations (Student’s *t*-test *p* = 0.009). In addition, one case of EBV-associated gastric cancer with no *PIK3CA* mutations had a mutation in the gene encoding for the regulatory subunit of PI3K, *PIK3R1*, instead. Both *ERBB2* and *PIK3R1* alterations would be predicted to activate the PI3K/AKT pathway, similar to *PIK3CA* mutations.

**Figure 5:**
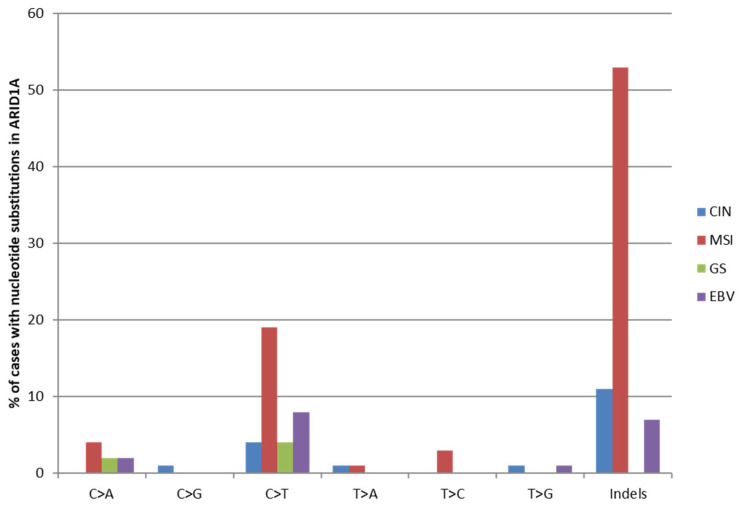
Percentage of cases with specific base substitutions in *ARID1A* according to the subtype of gastric adenocarcinomas. The conventional pyrimidine code is shown on the x-axis. CIN: Chromosome Instability subtype, MSI: Microsatellite Instability subtype, GS: Genomically Stable subtype, EBV: EBV-associated subtype, Indels: Insertions deletions. Data are from The Cancer Genome Atlas (TCGA).

*ARID1A* mutations were also frequent in EBV-associated gastric cancers, with 56.7% of the cases in this group possessing mutations in the gene. In this case, the MSI-high group displayed an even higher prevalence of *ARID1A* mutations, with 75.3% of samples having mutations. The pattern of mutations was different between *ARID1A* and *PIK3CA*, with 58.2% of *ARID1A* mutations across groups being insertions and deletions (indels), which were absent in *PIK3CA*, followed by C>T transitions in 28.7% of total *ARID1A* mutations. The percentage of indels and C>T transitions within the EBV-associated and the MSI-high groups also varied, with indels constituting 66.3% of mutations in MSI-high cancers and 38.9% of mutations in EBV-associated cancers (Student’s *t*-test *p* = 0.05, Figure 5), and C>T transitions contributing 23.8% of mutations in MSI-high cancers and 44.4% of mutations in EBV-associated cancers (Student’s *t*-test *p* = 0.08).

*BCOR*, a third gene with a mutation frequency in EBV-associated gastric cancers higher than in the whole gastric adenocarcinoma cohort (20% versus 8%), was also mutated in a high percentage (24.7%) of MSI-high gastric cancers. The underlying substitutions differed between the two groups, with C>T transitions constituting 50% (3 of 6 mutations) in EBV-associated cancers and 9.5% (2 of 21 mutations) in the MSI-high group (Student’s *t* test *p* = 0.05).

**Figure 6:**
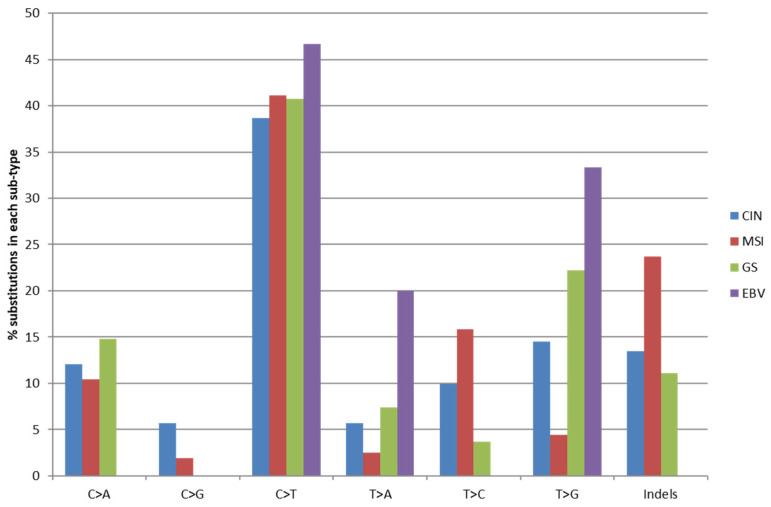
Percentage of cases with specific base substitutions in five prevalent cancer-associated genes in gastric cancer that are not frequently mutated in EBV-associated cancers (*TP53*, *LRP1B*, *FAT4*, *KMT2D*, and *KMT2C*), according to the subtype of gastric adenocarcinomas. The conventional pyrimidine code is shown on the x-axis. CIN: Chromosome Instability subtype, MSI: Microsatellite Instability subtype, GS: Genomically Stable subtype, EBV: EBV-associated subtype, Indels: Insertions- deletions. Data are from The Cancer Genome Atlas (TCGA).

Five other cancer-associated genes frequently mutated in gastric adenocarcinomas, *TP53*, *LRP1B*, *FAT4*, *KMT2D*, and *KMT2C*, were mutated less frequently in EBV-associated gastric cancers than in the entire cohort (Figure 1). The specific substitutions in these genes displayed some similarities and differences in the four gastric cancer subtypes (Figure 6). The most common substitutions in all subtypes were C>T transitions, constituting 38% to 46% of all mutations. Indels were absent in EBV-associated cancers, while they underlined 24% of the mutations in the five genes in MSI-high carcinomas and 11% to 14% in CIN and GS subtypes. Next to C>T transitions, T>G and T>A transversions were frequent in these five genes in EBV-associated cancers (Figure 6).

**Figure 7:**
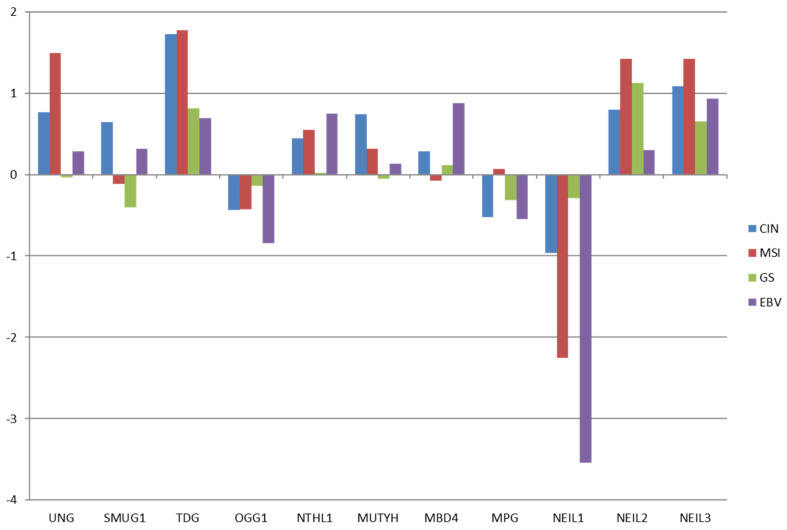
mRNA expression (mRNA expression z score relative to normal samples) of DNA glycosylases according to the genomic subtype of gastric cancers. CIN: Chromosome Instability subtype, MSI: Microsatellite Instability subtype, GS: Genomically Stable subtype, EBV: EBV-associated subtype. Data are from The Cancer Genome Atlas (TCGA).

DNA glycosylases are involved in Base Excision Repair (BER), which, in conjunction with translesion polymerases, repairs different focal lesions in DNA produced by exogenous and endogenous insults, resulting in substitutions due to the low fidelity of the process. Table 2 lists DNA glycosylases and translesion polymerases and describes their key functions. Among DNA glycosylases, *NEIL2* was the only one with genomic alterations in more than 5% (8.4%) of samples in the TCGA gastric cancer cohort, mostly copy number alterations (7.5%, Table 3). Mutations in 1 or more of the 11 glycosylases were present in 9.1% of the samples in the cohort, but only two mutations (one each in *NTHL1* and *MUTYH*) were classified as pathogenic, while the rest were of unknown significance. In the EBV-associated subtype, no mutations in glycosylases were observed in any of the 30 samples, and only 1 sample (3.3%) had copy number alterations, a deletion in *NEIL2*. At the mRNA level, NEIL1 showed significant down-regulation in EBV-associated gastric cancers, with less pronounced down-regulation of this glycosylase observed in MSI-high cancers (Figure 7). No other glycosylases were significantly (mean mRNA z score relative to normal samples above 1 or below −1) up-regulated or down-regulated in EBV-associated gastric cancers. This suggests that glycosylase deregulation is not a significant source of mutagenesis in EBV-associated gastric cancers.

**Figure 8:**
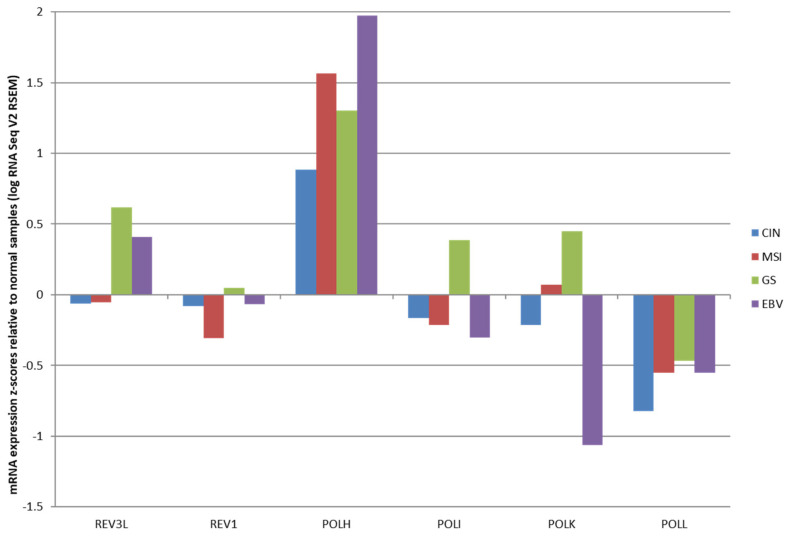
mRNA expression (mRNA expression z score relative to normal samples) of translesion polymerases (REV3L, REV1, POLH, POLI, POLK, and POLL) according to the genomic subtype of gastric cancers. CIN: Chromosome Instability subtype, MSI: Microsatellite Instability subtype, GS: Genomically Stable subtype, EBV: EBV-associated subtype. Data are from The Cancer Genome Atlas (TCGA).

**Figure 9:**
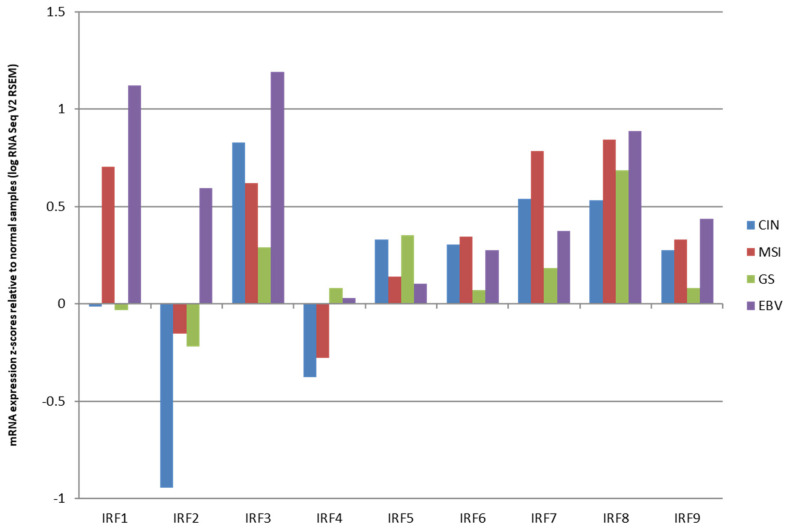
mRNA expression (mRNA expression z score relative to normal samples) of IRF family transcription factors (IRF1-9) according to the genomic subtype of gastric cancers. CIN: Chromosome Instability subtype, MSI: Microsatellite Instability subtype, GS: Genomically Stable subtype, EBV: EBV-associated subtype. Data are from The Cancer Genome Atlas (TCGA).

**Figure 10:**
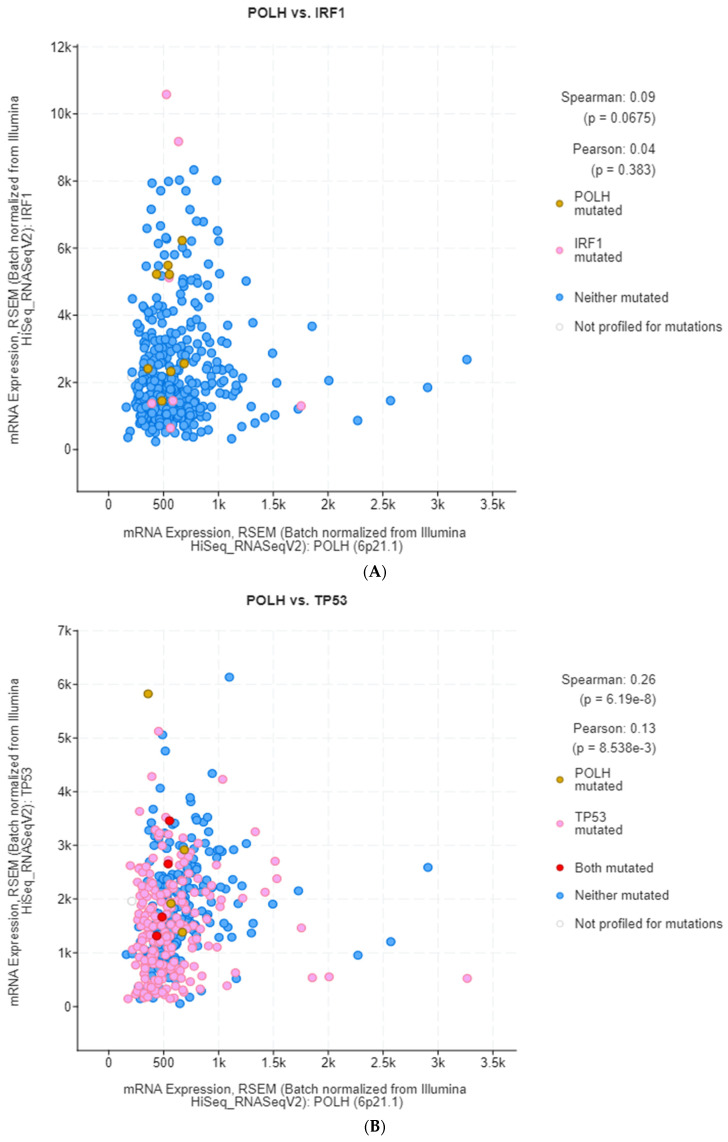
Correlation of mRNA expression (calculated with the RSEM algorithm) of polymerase η (POLH) (**A**) with the mRNA expression of IRF1, and (**B**) with the mRNA expression of p53 (gene *TP53*) in gastric cancers. Data are from The Cancer Genome Atlas (TCGA).

**Figure 11:**
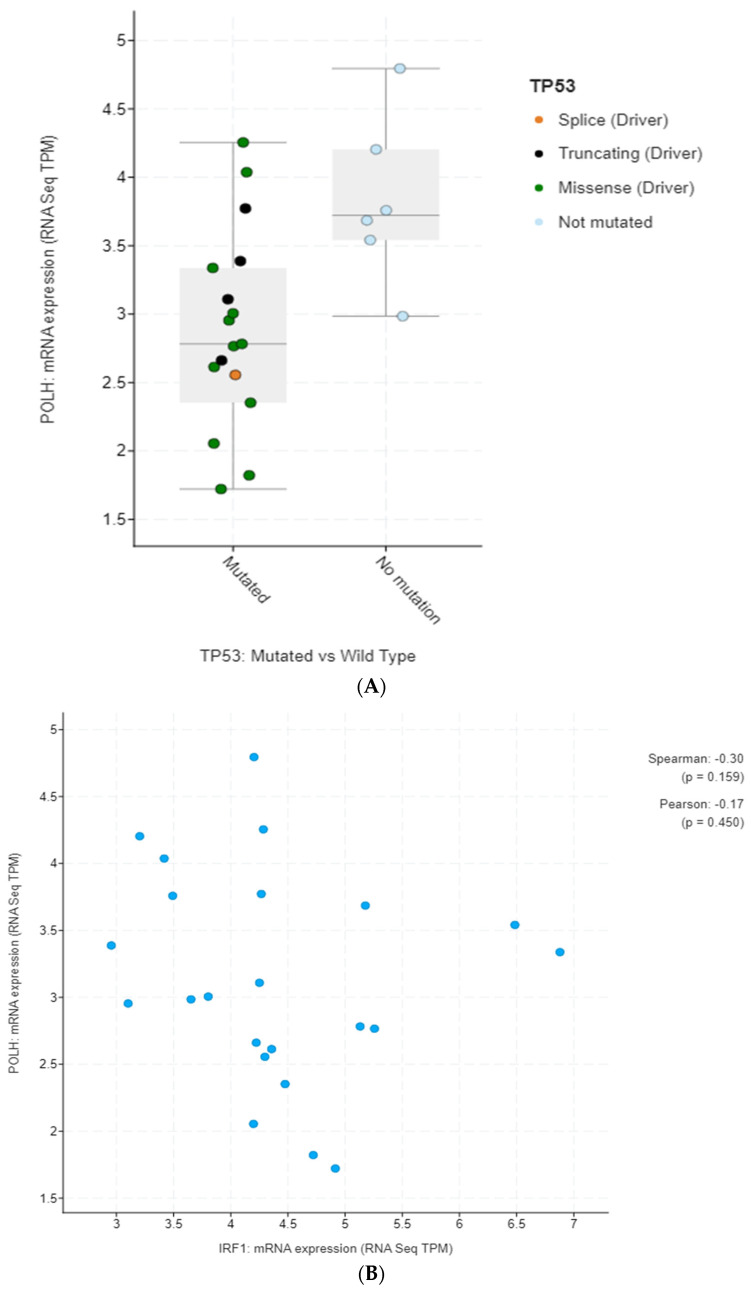
(**A**). Polymerase η (POLH) mRNA expression in gastric cancer cell lines with or without *TP53* mutations. (**B**). Correlation of mRNA expression of polymerase η (POLH) with IRF1 mRNA expression in gastric cancer cell lines. Data are from the Cancer Cell Line Encyclopedia (CCLE).

Next, potential causes of the increased prevalence of T to G transversions in EBV-associated gastric cancers were sought through scanning of COSMIC signatures with significant T>G components. Such signatures included SBS9, associated with polymerase η (POLH) somatic hypermutation activity, SBS17b, potentially associated with 5FU treatment, SBS88, associated with colibactin exposure from Escherichia coli carrying the pks pathogenicity island, and several signatures of unknown etiology (SBS28, SBS40b, SBS41, SBS89, SBS93, SBS96, SBS104, SBS106). Among the three known etiologies of signatures with significant T>G components, 5FU could be excluded as most patients in the cohort were untreated at the time of the biopsy, and persistent exposure to Escherichia coli was unlikely in the gastric epithelium. SBS17b signature may also be associated with gastric reflux, which, however, is not restricted to EBV-associated cancers [24]. The mRNA levels of Polymerase η encoded by POLH are up-regulated in EBV-associated cancers, and this up-regulation is higher than in other subtypes of gastric cancer (Figure 8, ANOVA *p* = 0.01). Other translesion polymerases were not significantly altered compared with normal samples (REV3L and polymerase ι) or were downregulated (polymerase κ, Figure 8). The expression of polymerase η protein was intermediate to low in most samples of stomach adenocarcinomas in the Human Protein Atlas (which did not categorize samples according to subtypes), compared with intermediate expression in normal gastric epithelium glands (Table 4). A similar analysis for REV3L, the catalytic unit of polymerase ζ (POLZ) and of polymerase ι (POLI), showed intermediate protein expressions in normal gastric epithelium and intermediate to low expression in most samples of gastric cancer for REV3L and low to absent expression for polymerase ι (Table 4). POLH up-regulation is mediated by transcription factors p53 and IRF1. Interestingly, p53 was rarely mutated in EBV-associated gastric cancers (Figure 1), while IRF1 was up-regulated specifically in those cancers compared with other gastric subtypes (Figure 9). MSI-high gastric cancers also showed a lower level of IRF1 up-regulation, which is absent in CIN and GS subtypes. Among the other IRF family members, IRF3 and IRF8 were also up-regulated in EBV-associated cancers, with the latter displaying up-regulation also in other subtypes (Figure 9). The mRNA expression of POLH was significantly positively correlated with the expression of TP53 and showed a trend for positive correlation with IRF1 in the entire gastric cohort (Figure 10). However, the correlation was not observed specifically in the EBV-associated sub-cohort, possibly because of small numbers and the dynamic regulation of mRNA expressions not optimally captured in the single-time mRNA evaluations (Corrected Spearman correlation coefficient q > 0.05). Gastric adenocarcinoma cell lines with *TP53* mutations had significantly lower expression of POLH than cell lines with wild type TP53 (Student’s *t* test *p* = 0.01, Figure 11A), while no correlation was observed between the expression of POLH and the expression of IRF1 (Pearson correlation co-efficient *p* = 0.45, Figure 11B), suggesting that a functional p53 is more important for the regulation of polymerase η expression in gastric cancers.

## 4. Discussion

EBV-associated gastric cancers constitute the least prevalent of the four gastric cancer subtypes identified by TCGA. Nevertheless, it is worth studying because of its intriguing pathogenesis, which may also be relevant for other virus-associated cancers that contribute to 10–20% of all human cancers [25,26]. Common themes in the pathogenesis of several viral cancers have been observed, including increased DNA methylation and low prevalence of *TP53* mutations [25].

Several important hints for the pathogenesis of EBV-associated gastric adenocarcinomas are derived from the data presented in the current analysis. C to T transitions resulting from the spontaneous deamination of methyl-cytosine were not increased in EBV-associated gastric cancers, despite the increased DNA methylation induced by EBV (the epigenetic hallmark of these cancers), suggesting that spontaneous deamination of methylated cytosines by the virus is not a direct, specific cause of increased mutagenesis. In contrast, these C to T transitions underlined a higher percentage of mutations in EBV-associated cancers in the specific case of genes that had increased prevalence of mutations in this subset, compared with other subtypes of gastric cancers (*PIK3CA*, *ARID1A*, and *BCOR*), suggesting that DNA methylation may be the cause of the increased prevalence of mutations in these particular gene cases, independently of the virus. These data argue for *PIK3CA* mutations predating the infection by EBV and being required for the development of malignancy. In this scenario, *PIK3CA* mutations act as the initiator event, and the EBV infection provides a selection event for the *PIK3CA*-mutated clones. Two facts arguing for this sequence of events are that no specific COSMIC signature associated with EBV has been identified and that in *PIK3CA* wild-type EBV-associated gastric cancers, alternative means of activating the PI3K/AKT pathway are often present, potentially serving as functional substitutes for *PIK3CA* mutations. In contrast to *PIK3CA* and *ARID1A*, other frequently mutated gastric cancer-associated genes, which are not more prevalent in EBV-associated cancers, did not show increased rates of C to T transitions compared with other subtypes.

The additional role of ARID1A mutations and the resulting SWI/SNF dysfunction in the sequence of pathogenic events of EBV-associated gastric cancers and the associated DNA hypermethylation is also of interest. A study of EBV-associated gastric carcinomas and adjacent non-neoplastic mucosa showed that ARID1A loss was present in both cancer and non-cancer mucosa cells, suggesting an early role in neoplastic transformation and facilitation of EBV-mediated cancer propagation [27]. The dysfunction of the SWI/SNF complex may affect normal nucleosome mobilization and lead to failure of glycosylases to access damaged DNA for base excision repair. The loss of ARID1A and SWI/SNF dysfunction promotes CpG island methylation and the CIMP phenotype and has been observed in EBV-associated gastric cancers and other non-EBV-associated cancers with CIMP, such as colorectal and endometrial carcinomas [28]. Methylation in CpG islands following knockout of *ARID1A* was observed in loci with pre-existing trimethylation in Lysine 27 of histone 3 (H3K27) and loci that acquired the trimethylation following *ARID1A* knockout. Therefore, it is plausible that *ARID1A* mutations precede EBV transformation and promote the CIMP phenotype, facilitating hypermethylation, in EBV-associated cancers. In contrast, in cases where the CIMP phenotype induces methylation in the promoter of MLH1, transformation is propagated along the lines of MMR deficiency, resulting from MLH1 loss of expression.

BCOR is an epigenetic regulator and member of the Polycomb Repressive Complex PRC1.1 non-canonical complex [29]. It is a co-repressor of the transcription regulator BCL6, and its alterations are present in a wide range of malignancies, including carcinomas, sarcomas, and hematologic cancers. PRC complexes are associated with transcription suppression and have functions opposing SWI/SNF transcription induction, and therefore BCOR mutations in combination with ARID1A mutations further deregulate the epigenetic landscape of EBV-associated cancers. The interplay of the other polycomb complex, PRC2, with SNF/SNF was shown in a study of tumors bearing *SMARCB1* alterations, which were specifically sensitive to an EZH2 inhibitor [30]. Moreover, EZH2 inhibition was synthetically lethal with *ARID1A* mutations, suggesting a broader synthetic lethality of EZH2 inhibition and SWI/SNF dysfunction [31]. Relevant for EBV-associated gastric cancers, the mechanism of synthetic lethality was de-repression of PI3K/AKT pathway inhibitor PIK3IP1. *BCOR* mutations have been associated with MYC overexpression in EBV-associated extranodal NK/T cell lymphoma, nasal type [32]. MYC has a role in suppression of the EBV lytic program in B cells, and down-regulation of MYC in plasma cells is associated with lysis [33]. However, MYC is not up-regulated in EBV-associated gastric cancers, and whether alternative suppressors of the EBV lytic program are regulated by BCOR remains to be shown. Both the high level of DNA hypermethylation observed and the alterations in epigenetic modifiers argue for the importance of epigenetic modifications in EBV-associated gastric cancers, acting in parallel with a low number of key additional genetic alterations, such as *PIK3CA* mutations. The importance of epigenetic modifications for cancer pathogenesis is a general theme that has been brought to the forefront of cancer research in recent years [34].

POL η (POLH), one of the several translesion polymerases involved in DNA replication at damaged sites, was up-regulated in EBV-associated gastric cancers. POL η plays a key role in replication stress recovery, preventing cell-cycle arrest and cell death [35]. Its expression is regulated by transcription factors IRF1 and p53 [36,37]. In EBV-associated gastric carcinomas, wild-type p53 may be involved in POL η up-regulation to secure cell survival. This is a paradoxical function for p53, which usually promotes cell-cycle arrest or apoptosis, but is part of the program for recovery of non-lethally damaged cells.

From a therapeutic point of view, EBV positivity is predictive of improved survival outcomes with chemo-immunotherapy treatment consisting of nivolumab immunotherapy plus chemotherapy [38]. The benefit was driven by tumors with PD-L1 Combined Positive Score (CPS) above five, while tumors with lower PD-L1 positivity or PD-L1 negative faired similarly with chemotherapy and chemotherapy/nivolumab [38]. In a large retrospective series of 766 patients from Asia, metastatic EBV-associated gastric cancers, as defined by EBER positivity, had a similar response to first-line fluoropyrimidine/platinum chemotherapy and a similar Progression Free Survival (PFS) compared with EBER-negative cancers (median PFS 6.4 months in EBV-positive and 6.7 months in EBV-negative tumors) [39]. However, there was a trend for better Overall Survival (OS) in patients with EBV-positive tumors (median OS 16.4 months versus 14 months in EBER-negative cancers) [39]. In an evaluation of 61 metastatic gastric cancer patients treated in a phase 2 trial of pembrolizumab in second line, all 6 EBV-positive patients showed a response to treatment (Overall Response Rate (ORR): 100%) [40]. PD-L1 CPS in the six patients ranged from 1 to 80, and the tumor mutation burden was low. MSI-high cancers showed an ORR of 85.7% in the trial. EBV-negative, microsatellite-stable (MSS) cancers had an ORR to pembrolizumab of 12% when p53 was wild type and 5% when it was mutated [40]. Loss of function of ARID1A in EBV-positive gastric cancers triggers an immune response by activating the cGAS/STING pathway, which promotes cytokine release and antigen presentation [41].

Besides immunotherapy, other therapeutic opportunities in EBV-associated gastric cancers include targeting the hypermethylated genome of the cancer cells, which is a prerequisite for the maintenance of the virus in the latency state. Hypomethylating cytidine analog drugs azacytidine and decitabine target both de novo and maintenance DNA methyltransferases and are used in hematologic malignancies [42]. However, the efficacy of these drugs in solid tumors has not been shown, and no approvals have been granted. This could be in part due to a non-targeted mode of development, which does not allow for the determination of specific molecular solid tumor sub-sets with potential sensitivity to these drugs. Moreover, the first-generation methyltransferase inhibitors, being non-specific, have a narrow therapeutic index. GSK3685032 is a next-generation demethylating agent targeting maintenance methyltransferase DNMT1 and sparing de novo methyltransferases DNMT3A and DNMT3B [43]. This specificity may provide an improved therapeutic index. A phase 1 trial of neo-adjuvant 5-azacytidine before EOX (Epirubicin, Oxaliplatin, and Capecitabine) chemotherapy in patients with gastric and gastroesophageal cancers, independent of subtype, showed that hypo-methylation of specific genomic marker loci was associated with response [44]. Overall, the regimen combining azacytidine with EOX produced three complete pathologic responses among the 12 patients included in the study, while five additional patients had partial responses. No published reports of more advanced development of this regimen are available, and the original report did not provide information regarding genomic tumor subtypes.

Given the high prevalence of *PIK3CA* mutations in EBV-associated gastric cancers, PI3K inhibitors could provide another opportunity to exploit therapeutically. However, attempts to target the PI3K/AKT/mTOR pathway in gastric cancer have not resulted in any drug successfully entering the clinic [45]. This has been due to a narrow therapeutic window of inhibitors targeting all isoforms of PI3K or combined inhibitors of PI3K and mTOR kinases, and resistance due to feedback activation of the pathway. Moreover, early development of most inhibitors was hampered by a lack of genomic guidance. A pre-clinical study of the mechanisms of resistance to PI3K inhibitor alpelisib (also known as BYL719) in *PIK3CA-mutated* cancers revealed that up-regulation of kinase PIM1, which has overlapping substrates with kinase AKT, contributed to resistance and co-treatment with a PIM inhibitor sensitized resistant cells [46]. Resistant cells were also sensitized to alpelisib by inhibition of BCL-xL in this model. This is particularly relevant in EBV-associated cancers, which show BCL2 family proteins over-expression.

In conclusion, several important observations are made in the current genomic analysis focusing on the small but distinct EBV-associated gastric cancer group and using a novel combined approach considering both the substitutions at the nucleotide level and the specific genes and codon locations. First, despite the highest methylation rate amongst any cancer, EBV-associated gastric carcinomas do not possess an increased percentage of C>T transitions compared with other subtypes. These transitions are the most common of all six single-nucleotide substitutions in all gastric cancer subtypes and in other cancers. Second, activation of the PI3K pathway plays a key role in creating a permissive environment for EBV-associated cancer promotion, as suggested not only by the high rate of *PIK3CA* mutations but also by other alterations activating the pathway in cancers without *PIK3CA* mutations. In contrast, *TP53* remains wild-type in EBV-associated gastric cancers and may play a key role in the upregulation of polymerase η, which shapes the nucleotide substitution signature of these cancers by increasing T>G transversions. The high prevalence of *PIK3CA* mutations and the absence of TP53 alterations are also observed in other virus-related carcinomas, such as uterine cervical cancers, and therefore may reflect broader pathogenic landscapes of viral carcinogenesis [47]. Therapeutic opportunities are suggested by all these observations.

## Figures and Tables

**Table 1:** Databases used in this study and electronic addresses.

Database	Electronic Address	Last Accessed Date
cBioportal	www.cbioportal.org	13 May 2026
COSMIC	www.cancer.sanger.ac.uk/signatures/	14 May 2026
The Human Protein Atlas	Proteinatlas.org	14 May 2026
OncoKb	www.oncoKb.org	17 May 2026

**Table 2:** DNA glycosylases and translesion polymerases: official symbols and function.

Symbol	Gene Name	Function
Glycosylases		
MPG	N-methylpurine DNA glycosylase	DNA glycosylase involved in base excision repair (BER)
OGG1	8-oxoguanine DNA glycosylase	Excises 8-oxoguanine, a by-product of exposure to reactive oxygen species (ROS)
NTHL1	Nth-like DNA glycosylase 1	DNA glycosylase with activity for oxidized pyrimidine residues has also apurinic/ apyrimidinic lyase activity
MUTYH	MutY homolog DNA glycosylase	Excises adenine when inappropriately paired with cytosine, guanine, or 8-oxo-7,8-dihydroguanine
SMUG1	Single-strand selective monofunctional uracil DNA glycosylase 1	Excises uracil inappropriately incorporated in DNA
MBD4	Methyl CpG Binding Domain 4	Binds to methyl CpG and is involved in DNA repair through its mismatch-specific glycosylase domain
TDG	Thymine DNA glycosylase	Excises mispaired thymine from DNA
UNG	Uracil DNA glycosylase	Excises uracil inappropriately incorporated in DNA
NEIL1	Nei like DNA glycosylase 1	DNA glycosylase involved in BER by excising oxidized pyrimidines
NEIL2	Nei like DNA glycosylase 2	DNA glycosylase involved in BER by excising 5-hydroxyuracil and 5-hydroxycytosine
NEIL3	Nei like DNA glycosylase 3	DNA glycosylase is involved in BER by excising oxidized DNA bases
Polymerases		
REV3L	REV3, like DNA-directed polymerase zeta catalytic subunit	Functions in translesion DNA synthesis
REV1	REV1 DNA-directed polymerase	Scaffold for translesion DNA polymerases
POLH	DNA polymerase eta	Participates in bypassing UV-damaged DNA, specifically thymine dimers
POLI	DNA polymerase iota	Participates in DNA synthesis across damaged template sites
POLK	DNA polymerase kappa	Participates in DNA synthesis across damaged template sites
POLL	DNA polymerase lambda	Participates in DNA extension during repair and plays a role in non-homologous end joining repair

**Table 3:** Alterations of DNA glycosylases in the entire TCGA gastric cancer cohort and in the genomic sub-groups. One mutation each in NTHL1 and MUTYH1 was classified as pathogenic; the rest were of unknown significance. CIN: Chromosomal Instability, CNA: Copy Number Alterations, MSI: Microsatellite Instability, GS: Genomically Stable, EBV: Epstein–Barr virus-associated gastric cancers.

	Entire (*n* = 440)		CIN (*n* = 223)		MSI (*n* = 73)		GS (*n* = 50)		EBV (*n* = 30)	
Glycosylase	Mutations (%)	CNA (%)	Mutations (%)	CNA (%)	Mutations (%)	CNA (%)	Mutations (%)	CNA (%)	Mutations (%)	CNA (%)
MPG	5 (1.1)	10 (2.3)	2 (0.9)	8 (3.6)	3 (4.1)	1 (1.4)	0	1 (2)	0	0
OGG1	3 (0.7)	6 (1.4)	1 (0.4)	4 (1.8)	0	2 (2.7)	0	0	0	0
NTHL1	5 (1.1)	4 (0.9)	1 (0.4)	3 (1.3)	3 (4.1)	0	1 (2)	1 (2)	0	0
MUTYH	7(1.6)	2 (0.5)	1 (0.4)	2 (0.9)	5 (6.8)	0	0	0	0	0
SMUG1	3 (0.7)	6 (1.4)	0	3 (1.3)	3 (4.1)	0	0	1 (2)	0	0
MBD4	5 (1.1)	2 (0.5)	1 (0.4)	2 (0.9)	2 (2.7)	0	0	0	0	0
TDG	2 (0.5)	2 (0.5)	0	1 (0.4)	2 (2.7)	0	0	0	0	0
UNG	2 (0.5)	1 (0.2)	1 (0.4)	1 (0.4)	1 (1.4)	0	0	0	0	0
NEIL1	6 (1.4)	5 (1.1)	0	4 (1.8)	5 (6.8)	0	0	0	0	0
NEIL2	4 (0.9)	33 (7.5)	1 (0.4)	24 (10.8)	3 (4.1)	1 (1.4)	0	3 (6)	0	1 (3.3)
NEIL3	8 (1.8)	10 (2.3)	1 (0.4)	7 (3.1)	5 (6.8)	2 (2.7)	0	0	0	0
total	40 (9.1)	81 (18.4)	9 (4)	59 (26.5)	32 (43.8)	6 (8.2)	1 (2)	6 (12)	0	1 (3.3)

**Table 4:** Expressions of translesion polymerases in normal gastric epithelium and gastric cancers at the mRNA and protein level. Data for mRNA in normal tissues were from GTEx (Genotype-Tissue Expression project). Data for mRNA in cancer were from TCGA (The Cancer Genome Atlas). Protein data were from the Human Protein Atlas. nTPM: Normalized transcripts per million. FPKM: Fragments per Kilobase of exon per Million reads. NA: Not available.

	Normal Tissue		Cancer	
	mRNA (Median nTPM)	Protein	mRNA (Average FPKM)	Protein
REV3L	4.3	Medium (HPA064853)	8.8	High: 0 of 10/medium: 4 of 10/low: 3 of 10/none: 3 of 10 (HPA064853)
REV1	7.8	Medium (HPA044534) Medium (HPA051036)	4	High: 0 of 11/medium: 8 of 11/low: 2 of 11/none: 1 of 11 (HPA044534), High: 0 of 12/medium: 1 of 12/low: 2 of 12/none: 9 of 12 (HPA051036)
POLH	3.2	Medium (HPA006721) Medium (HPA026762)	9.2	High: 0 of 12/medium: 2 of 12/low: 3 of 12/none: 7 of 12 (HPA006721), High: 0 of 12/medium: 4 of 12/low: 7 of 12/none: 1 of 12 (HPA026762)
POLI	9.1	Medium (HPA064696)	3.2	High: 0 of 11/medium: 0 of 11/low: 2 of 11/none: 9 of 11 (HPA064696)
POLK	3.3	NA	2.3	NA
POLL	25.9	NA	12.7	NA

## Data Availability

The original contributions presented in this study are included in the article. Further inquiries can be directed to the corresponding author(s).
